# Increasing competition for water resources in the food and energy industries

**DOI:** 10.1371/journal.pone.0312836

**Published:** 2024-11-25

**Authors:** Xian Liu

**Affiliations:** 1 School of Geography Sciences, Shanxi Normal University, Taiyuan, China; 2 Ecological Environment Research Center of Middle Yellow River, School of Geography Sciences, Shanxi Normal University, Taiyuan, China; Central Queensland University, AUSTRALIA

## Abstract

Exploring the mechanisms of competition for water resources in the regional food and energy industries,taking effective countermeasures in advance will help to promote the sustainable development of the regional food and energy industries. Based on the theory of water footprint, this paper quantifies the water footprint of grain and energy in Shanxi Province, China, and the competition index of energy-grain industry to water resources. Through the ratio of grain blue water and energy water footprint to total water consumption in the region, this study coupled the characteristics of their proportional changes to obtain a competition index that can characterize the competition relationship between regional food and energy industries for water resources. The results showed that (1) In 2000–2021, although the grain yield increased by 81.1%, the grain water footprint only increased by 11.2%, with an average annual growth of 1.0%. The proportion of blue, green and gray water footprints changed from 14.1%, 54.3% and 31.6% in 2000 to 18.8%, 41.2% and 39.9% in 2021, respectively. (2) The energy water footprint increased at an average annual rate of 10.8%, an increase of 544.7% over the period. The coal, natural gas and thermal water footprints in 2021 have increased by a factor of 2.7, 109.2 and 4.0, respectively, compared to 2000. Coal’s share of the energy water footprint is 70.6%. (3) The average value of the food and energy industry’s competition index for water resources is 0.77, increasing from 0.42 in 2000 to 0.94 in 2021, an average annual increase of 4.3%. These results show that the future sustainable development of the region is facing the major challenge. Therefore, from the perspective of reducing the demand for crop and energy water footprint, some suggestions are put forward to effectively promote the healthy development of the region.

## Introduction

Water security is a cornerstone for healthy regional development and an irreplaceable resource for sustainable agricultural and energy production [1]. Both the production processes of food and energy require water inputs, and water security leads to synergies and competition between food and energy, directly affecting regional food and energy security [2]. Against the backdrop of global warming, the nexus between water, energy and food has formed a mutually constraining relationship that is highly sensitive and vulnerable [3–5]. The synergies and trade-offs among the three not only help to mitigate regional water crises, but also allow for early detection and effective policy interventions to alleviate conflicts [3].

China’s food, energy and water issues are the concern to residents. China is the world’s largest producer and consumer of food and fuel and is also experiencing a national water crisis [6]. It is also one of the countries with relatively scarce water resources, with per capita water resources amounting to only 25% of the global level [7]. Based on the such natural resource conditions, China faces a number of water and energy problems [8–11]. In order to effectively promote synergistic regional agricultural and economic development, the Chinese Government has established a strict water resource management system [12–16], and the rapid economic growth requires energy to provide fuel [17, 18]. Therefore, clarifying the characteristics of the food and energy industries’ competition for limited water resources will help to alleviate regional water stress and provide a basis for accelerating high-quality regional development and effective countermeasures.

A great deal of research has been conducted on the synergistic mechanisms between water, energy and food, and numerous scholars have recognized the importance of all three in high-quality regional development [19]. Malik first studied the relationship between water and energy interactions in India [20]. In the following years, more and more scholars began to pay attention to the complex relationship between water and energy, and the scale of research showed a diversified trend [21–23]. In the twenty-first century, explosive population growth has increased pressure not only on water and energy supplies, but also on the demand for food [24, 25]. Scholars have shifted their focus from the water-energy nexus to the water-energy-food nexus [26–29]. Water resource was the core element in the water-energy-food triad, which affects the efficiency, stability and sustainability of the water-energy-food system, but there were fewer research about water resource evaluation in the water-energy-food triad and focused on the synergistic and trade-off development of water resource and other elements, which was insufficient in terms of research depth and breadth. Some scholars have focused on the interrelationships between water resources, food and energy production, exploring the positive and negative feedback mechanisms of water resources development potential on the nexus [30]. For the water resource elements in the water-energy-food system, they used global optimization methods and other methods to simulate and predict water resources [30]. A framework for analyzing the existence of food systems in the nexus relationship and a coupled coordination evaluation index system were solved, and a quantitative evaluation of the development of synergies and trade-offs in the nexus relationship was carried out. In addition, the introduction of the water footprint theory based on the water-energy-food system perspective has broadened the scope of water resources management, providing an effective means to effectively quantify the utilization efficiency of water in the crop production process and to assess its evolutionary characteristics under the conditions of different factors, such as time, space, and types of water sources [31–34]. Introducing the water footprint into the water-energy-food coupling relationship has certain advantages for quantifying the competitive mechanisms and assessing the evolutionary trends of water consumption characteristics and spatio-temporal patterns in food and energy production [35, 36]. In summary, the current research on the characteristics of the water-energy-food coupling relationship mainly focuses on the mechanism level. The results of introducing the water footprint concept into the coupled water-energy-food system and quantitatively characterizing the competition among the three are fewer, especially for Shanxi Province, where the competition between China’s energy and food industries is extremely intense. In 2019, the per capita water resources in Shanxi province was only 261.31 m^3^, which was 1/8 of China’s average and 1/24 of the world’s average. Shanxi is the most important coal energy base in China, with a raw coal production of 1.01 billion tons in 2020, which accounts for 25.9% of the national total production. As a large province of coal outward transfer, Shanxi province coal outward transfer volume of 1.06 billion tons, accounting for about 1/3 of the country. Affected by the topography, natural resources and other factors, Shanxi province food production conditions are more fragile, the food gap is larger. Under the background of shortage of water resources and unreasonable energy consumption structure, scientific analysis of water-energy-grain interaction in Shanxi Province is of great significance to the sustainable development of the region.

Based on the shortcomings of existing studies, this study takes Shanxi Province, where the food and energy industries compete fiercely for water resources, as the study area. On the basis of quantifying the water footprint of the production of major grain crops in Shanxi Province, the spatial and temporal evolution of the water footprint of crop production is explored. Quantify the energy-water footprints of major energy products and analyze their change characteristics under long time series conditions. On this basis, further analyze the change characteristics of the energy and food industries’ competition index for regional water resources, and reveal the competitive relationship or mechanism of the energy and food industries for water resources. Based on the results of the study, effective suggested measures to promote the healthy development of the region are proposed. This study can provide a theoretical basis for improving the high-quality development of the regional agricultural and energy industries, as well as the healthy and sustainable development of the region.

## Materials and methods

### Overview of the study area

**Fig 1:**
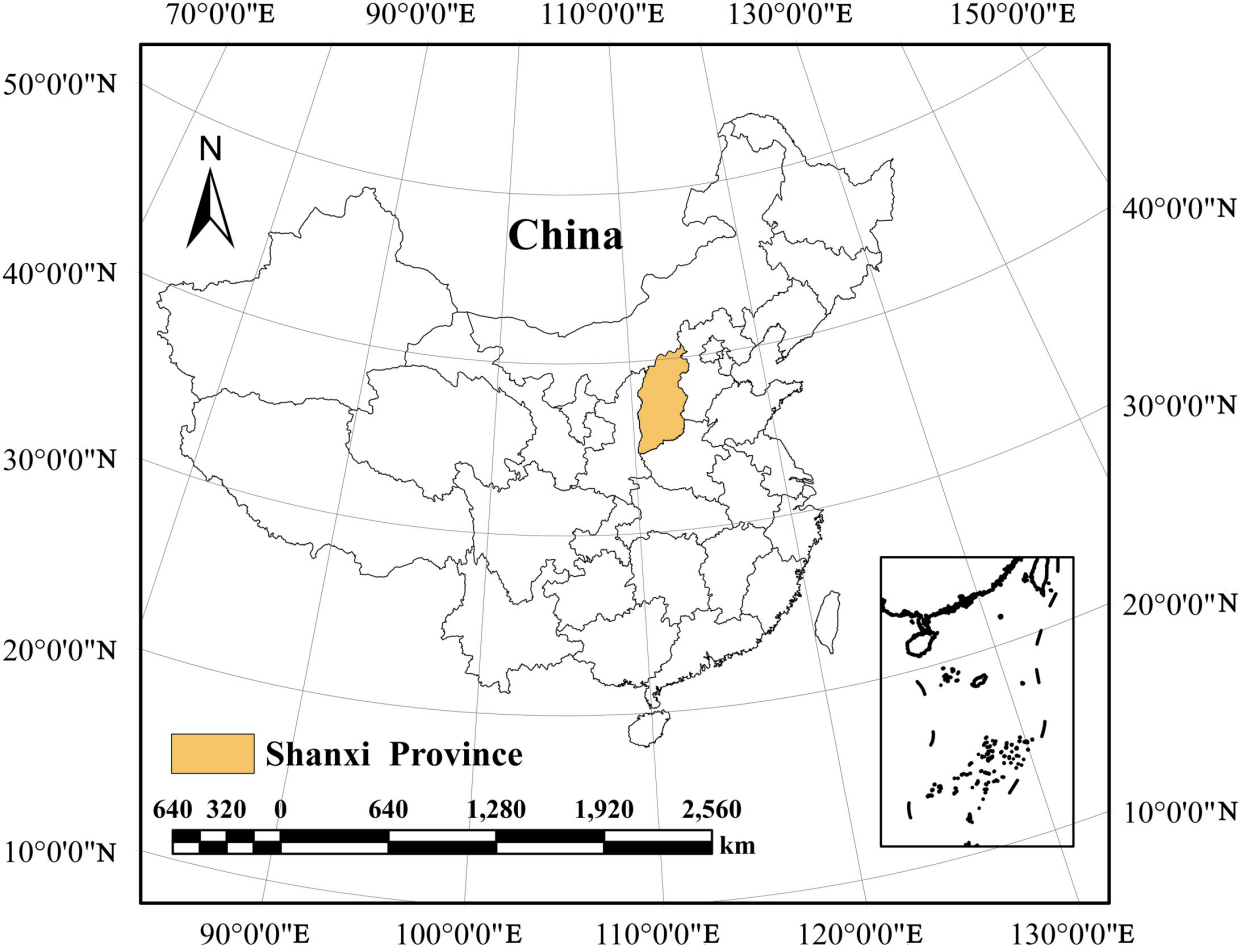
Geographical location of the study area (The shapefiles is available from standard map service, URL: http://bzdt.ch.mnr.gov.cn/).

Shanxi Province (34°34′—40°44′ N, 110°14′—114°33′ E), as an important province in guaranteeing China’s food and energy supply security, has an average annual precipitation of only 470 mm, and its per capita water resources possession is only 14% of the national level (Fig 1). Shanxi has been one of the most drought-hit provinces in China. Even so, in order to guarantee national food security, Shanxi Province has been engaged in high-intensity agricultural production, which has been occupying a large amount of water resources in the region. For example, in 2021, agricultural water consumption in Shanxi was 4.08 billion m³, accounting for 56.2% of the total water consumption. In a ranking of coal reserves, China is third in the world after the USA and Russian [37]. Shanxi is a major coal province in China. Its coal reserves rank second in China, accounting for 25% of China’s coal resources [37, 38]. Shanxi’s coal production has totaled 13 billion tons since 1949. 9 billion tons of coal have been transferred out of the province among that, accounting for more than a quarter of the country’s total production and more than 70% of China’s net inter-provincial coal transfers, making a great contribution to China’s economic and social development.

### Methods

#### Water footprint of crop production

$$WF_{G}\text{=}BWF+GWF\text{+}G^{’}WF$$ (1)

where, *WF*_*G*_ is the grain water footprint, m^3^. *BWF* is the blue water footprint (irrigation water consumption) of the food production process, m^3^.*GWF* is the green water footprint (precipitation consumption) of the food production process, m^3^.*G’WF* is the gray water footprint (the amount of water required to dilute pollutants from the agricultural production process), m^3^.

$$BWF\text{=}\sum_{c=1}^{n}\left(I_{G}^{c}\times A_{G}^{c}\right)$$ (2)

where, $I_{G}^{c}$ is the amount of water used for irrigation per unit area for food crop *c*, m^3^/ha. $A_{G}^{c}$ is the sown area of grain crop *c*, ha. *n* is the sum of all grain crop types.

$$GWF\text{=}\sum_{c=1}^{n}\left(W_{G}^{c}\times A_{G}^{c}\right)$$ (3)

where, $W_{G}^{c}$ is the green water consumption of grain crop *c* during the reproductive period, m^3^/ha.

$$W_{G}^{c}=10\text{min}(ET_{c},P_{e}^{c})$$ (4)

where, $P_{e}^{c}$ is the effective amount of precipitation for food crop *c* during the reproductive period, mm. *ET*_*c*_ is the actual evapotranspiration of the crop during the growing season, mm.

The average of the same daily precipitation of each station in the same region and in the same period is taken as the daily precipitation value of the same period in the region. Effective precipitation during the growth period of food crops was calculated using the method recommended by the Soil Conservation Service of USDA [39], as shown in Eq (5):

$$\begin{array}{l} P_{\text{e}}\text{=}\left\{\begin{array}{l} \frac{P(4.17-0.2P)}{4.17}\;(P<8.3)\\ 4.17\text{+}0.1P\;(P\geq 8.3) \end{array}\right\}\; \end{array}$$ (5)

where, *P* and *P*_*e*_ are the daily precipitation and effective precipitation, respectively, mm.

$$G^{’}WF\text{=}\frac{\alpha \times \text{AR}}{c_{\text{max}}-c_{nat}}\cdot A_{G}^{c}$$ (6)

where, *AR* is nitrogen fertilizer application amount per hectare, kg/ha.*α* is the leaching rate, generally 10%. *c*_max_ is the maximum allowable concentration, kg/m^3^. *c*_*nat*_ is the natural background concentration of the pollutant, and its value is 0 [40].

#### Calculation method of energy water footprint

**Fig 2:**
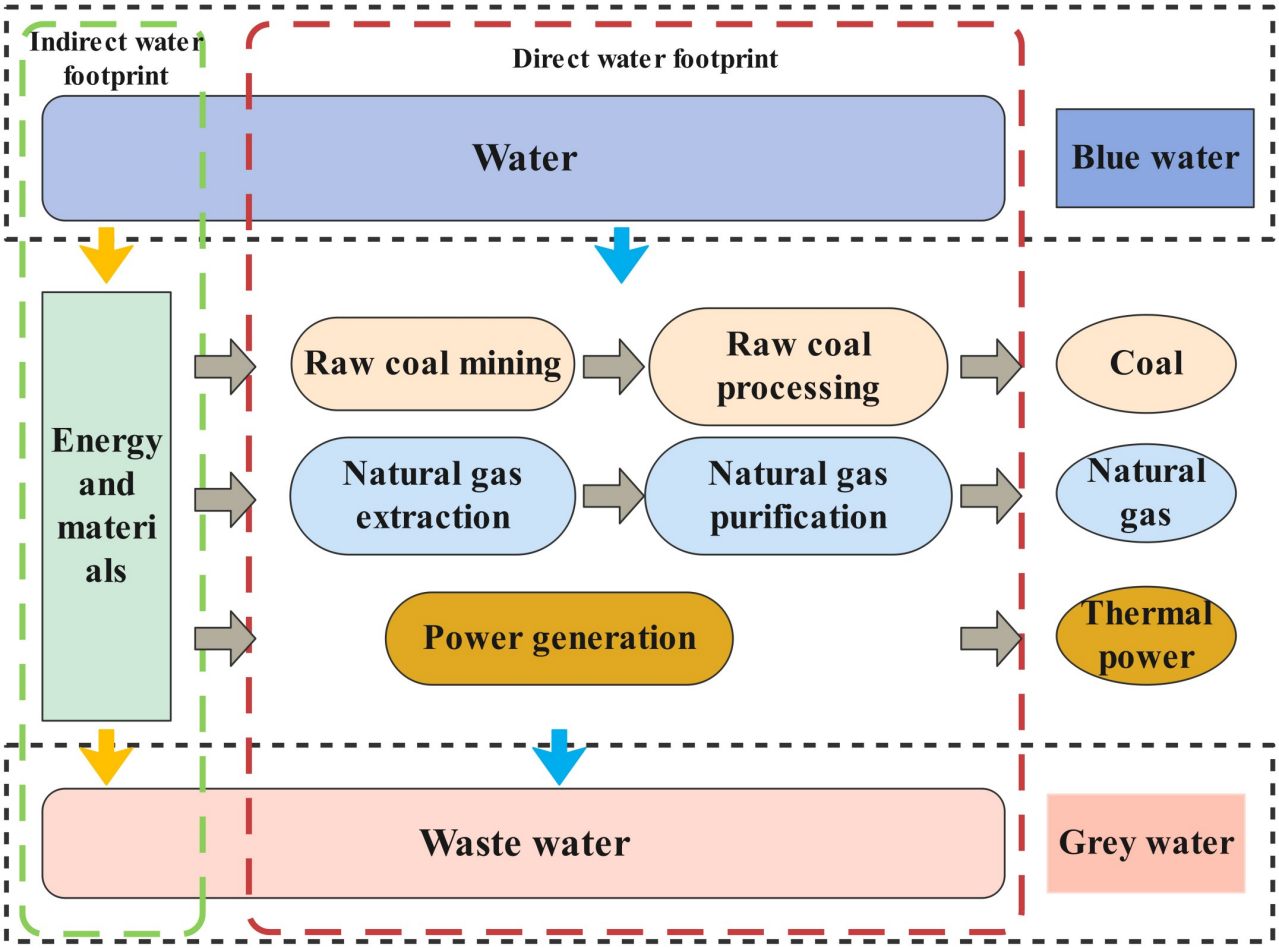
System boundaries of the energy-water footprint.

Coal, natural gas and thermal power, which use a large amount of water in the energy industry and are closely related to economic development and residents’ life, are selected for analysis. The energy production process includes both direct water use (water used in the energy production process) and indirect water use (water used in relation to inputs such as materials, energy and other resources) [41]. The energy water footprint of this study includes only blue and gray water and excludes green water, which is usually defined as the amount of rainwater consumed for agricultural production (Fig 2).


$$WF_{E}=WF_{E,\;direct}+WF_{E,\;indirect}=WF_{E,\;b,d}+WF_{E,\;g,d}+WF_{E,\;b,in}+WF_{E,\;g,in}$$ (7)

where, *WF*_*E*_ represents the water footprint of energy products (including coal, natural gas and thermal power generation), m^3^. *WF*_*E*,*direct*_ represents the direct water footprint in the production of energy products, m^3^. *WF*_*E*,*indirect*_ represents the indirect water footprint in the production of energy products, m^3^. *WF*_*E*,*b*,*d*_ represents the blue water footprint directly generated by the production process of energy products, m^3^. *WF*_*E*,*g*,*d*_ represents the gray water footprint directly generated during energy production, m^3^. *WF*_*E*,*b*,*in*_ represents the indirect blue water footprint of the energy product production process, m^3^. *WF*_*E*,*g*,*in*_ represents the indirect grey water footprint of the energy product production process, m^3^.

Taking into account all life cycle processes, the water footprint of an energy system should be the sum of the water footprint of each life cycle stage, expressed by the following formula:

$$WF_{E}=\sum_{i=1}^{n}\left(WF_{i(b,d)}+WF_{i(g,d)}\right)\cdot D_{i}+\sum_{j=1}^{n}\left(WF_{j(b,in)}+WF_{j(g,in)}\right)\cdot D_{j}$$ (8)

where, *WF*_*i(b*,*d)*_ represents the blue water footprint in the *i* production process, m^3^/GJ. *WF*_*i(g*,*d)*_ refers to the gray water footprint in the *i* production process, m^3^/GJ. *i* mainly represents the process of extracting and treating energy products.*WF*_*j(b*,*in)*_ refers to the blue water footprint of the *j*-th category of energy inputs, m^3^/GJ. *WF*_*j(g*,*in)*_ refers to the gray water footprint of the *j*-th category of energy inputs, m^3^/GJ. *D* is the heat of energy production, GJ. With regard to the specific processes of energy production and the resource inputs to each process, reference is made to the findings of Ding et al. [41].

The grey water footprint refers to the amount of water required to dilute the polluted water in the process of energy production to the discharge standard, calculated by formula (9) [40].

$$WF_{g,d}=\frac{L\times V_{p}}{C_{\text{max}}-C_{nat}}$$ (9)

where, *L* is the amount of wastewater produced by the production unit of energy products, m^3^/GJ. *V*_*p*_ is the amount of pollutants in the discharged wastewater, mg/m^3^. *C*_max_ is the maximum acceptable pollutant concentration in water, mg/m^3^; *C*_*nat*_ is the concentration of pollutants in natural water, mg/m^3^.

Various pollutants are emitted in the process of energy production, so the chemical oxygen demand (COD) with the largest pollutant emission is selected as the calculation index of gray water footprint [41]. The basis for the determination of *C*_max_, For coal, according to the Coal Industry Pollutant Emission Standard, the maximum COD value is 70 mg/L [32]. For natural gas and thermal power, this value is defined as 30 mg/L [32]. Due to the lack of data, the natural background concentration *C*_*nat*_ is usually set to 0.

#### Competition index of energy-food to water resources

$$I_{G}\text{=}\frac{BWF}{W_{A}}$$ (10)

$$I_{E}\text{=}\frac{WF_{E}}{W_{A}}$$ (11)

$$I\text{=2}\sqrt{I_{G}\times I_{E}}$$ (12)

where, *I*_*G*_ is the ratio of regional grain blue water footprint to total water consumption. *W*_*A*_ represents the total amount of regional water use, m^3^.*I*_*E*_ represents the ratio of regional energy water footprint to total water consumption. *I* is the competition index of food and energy industry to regional water resources.

#### Data sources

The meteorological data used in this study are from the China Meteorological Data Service Center (http://data.cma.cn). Agricultural data (e.g., grain yield, grain sown area, irrigation area and irrigation water consumption), energy data (coal, natural gas and thermal power generation) and water resources data were taken from the Shanxi Province and China Statistical Yearbook, Shanxi Province Water Resources Bulletin, China Water Resources Bulletin, China Rural Statistical Yearbook, China Agricultural Statistical Yearbook, China Environmental Statistical Yearbook, China Energy Statistical Yearbook, Coal and Electricity Base Development and Water Resources Research and China Coal-to-Liquid Development Status and Trend Analysis from 2001 to 2021.

## Results

### Analysis of historical evolution characteristics of grain water footprint

**Fig 3:**
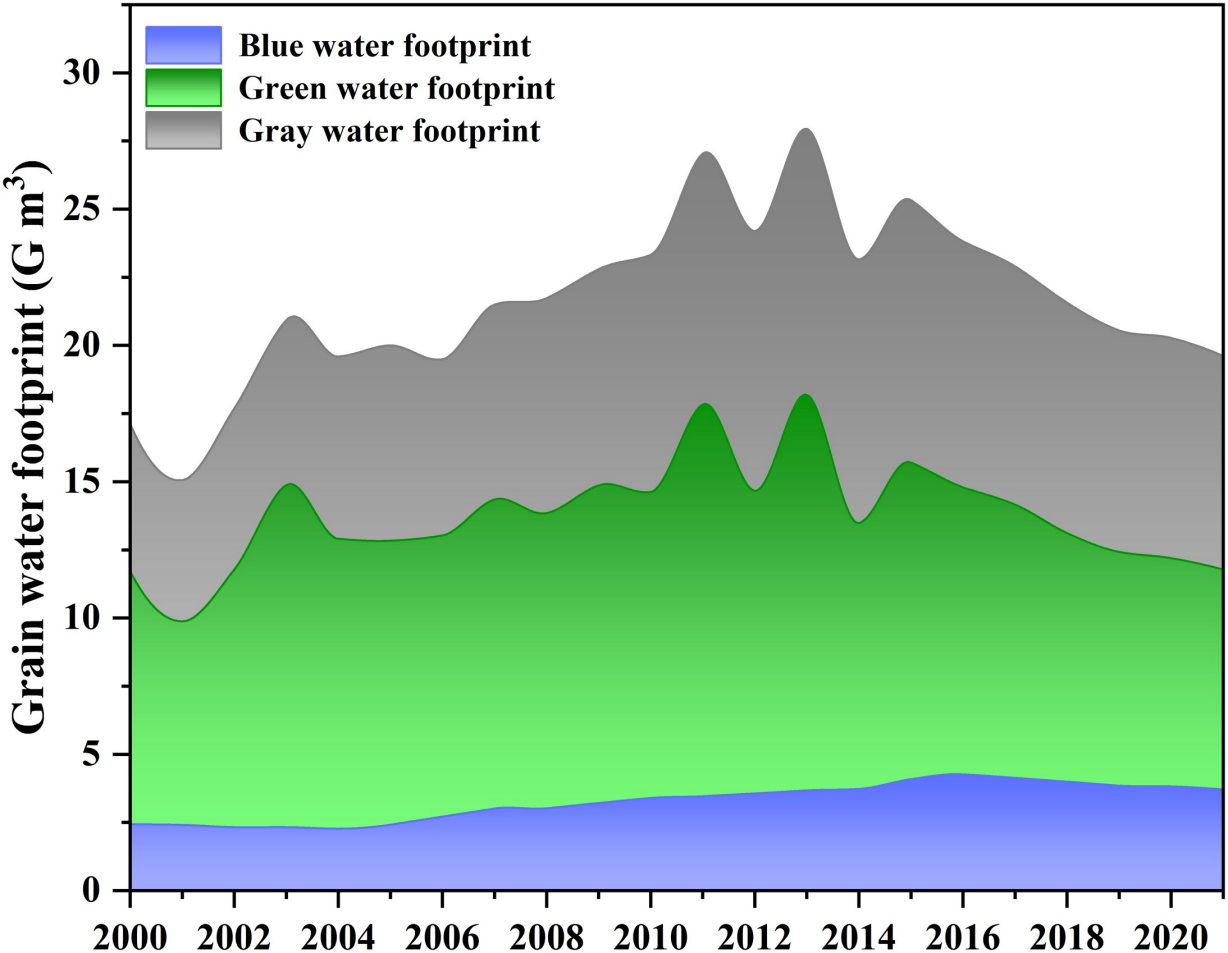
Trend of grain water footprint over time from 2000 to 2021.

The grain water footprint of Shanxi Province showed a trend of increasing first and then decreasing. From 2000 to 2021, on the basis of an increase of 81.1% in grain production in Shanxi Province, the grain water footprint only increased by 11.2%, with an average annual increase of 1.0% (Fig 3). The whole change process showed a significant increasing trend (*p*<0.01). The grain water footprint reached its maximum in 2013 (13.1%, 51.9% and 34.9% for blue, green and gray water footprints, respectively), which is 88.6% more compared to 2001. The structure of grain water footprint also shifted over the study period, with the share of blue, green and gray water footprints shifted from 14.1%, 54.3% and 31.6% in 2000 to 18.8%, 41.2% and 39.9% in 2021, respectively. Therefore, the water footprint of grain in Shanxi Province has been dominated by green and gray water footprints, in which the proportion of blue and gray water footprints has been increasing year by year, and the proportion of green water footprint is decreasing. For the blue water footprint, which was extremely close to the regional water supply and demand balance, it showed a significant increasing trend (*p*<0.01) from 2000 to 2021, with an average annual increase of 2.2%. The increase in the blue water footprint of food will undoubtedly exacerbate water stress and pose a challenge to the sustainability of regional industries.

**Fig 4:**
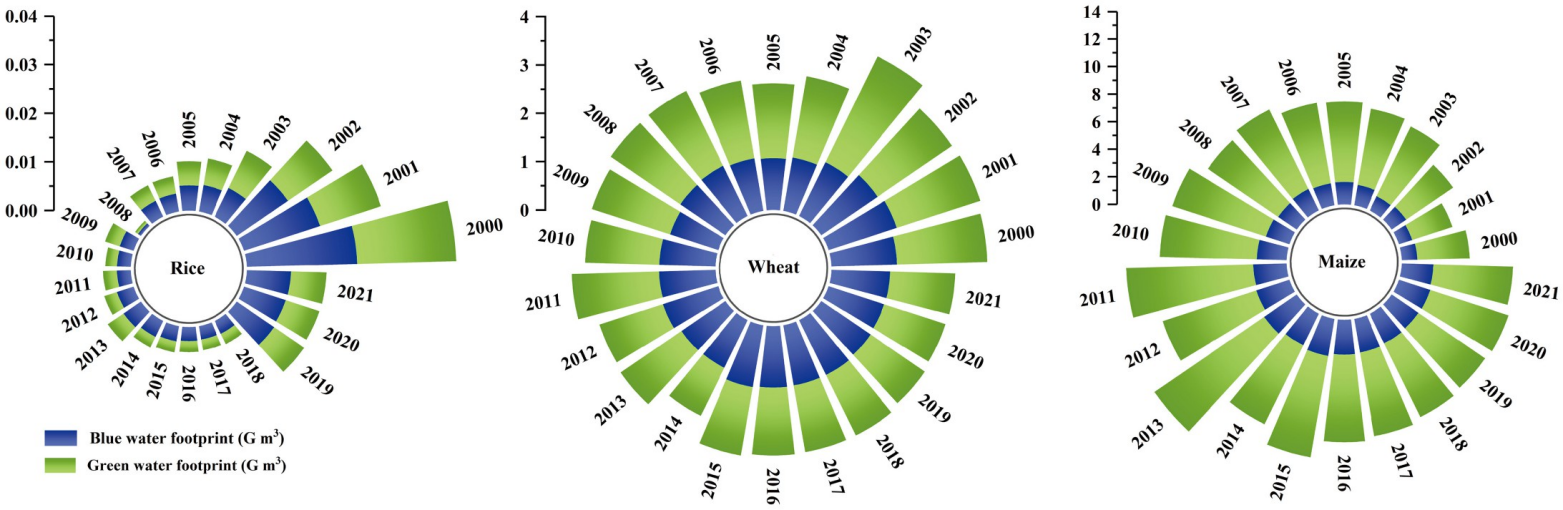
Structural characteristics of blue and green water for the three major food crops.

For the three major grain crops, Shanxi Province was dominated by maize and wheat. From 2000 to 2021, the blue and green water footprints of maize in Shanxi Province showed a significant increasing trend (*p*<0.01), of which the blue water footprint increased by 104.0%, with an average annual increase of 4.1% (Fig 4). The green water footprint increased by 52.2%, with an average annual increase of 3.7%. The percentage of multi-year blue and green water footprints for maize was 24.1% and 75.9%, which showed that precipitation played a crucial role in maize production in Shanxi Province. By analyzing the characteristics of the evolution of the share of the blue water footprint of maize over time, it can be seen that the share of its blue water footprint shows an increasing trend year by year, from 22.8% in 2000 to 28.6% in 2021. As the most important grain crop in Shanxi Province, blue water played an increasingly important role in the production of maize, which will inevitably increase the demand for regional water resources. For wheat, another major food crop, the blue water footprint and green water footprint showed an insignificant and significant decreasing trend from 2000 to 2021 (*p*<0.05), in which the blue water footprint decreased by 10.3% and the green water footprint decreased by 28.0%. There was little difference in the proportion of blue water footprint and green water footprint in wheat, and the average value was 43.2% and 56.8%, respectively. It can be seen that wheat production in Shanxi mainly depended on irrigation and precipitation. The proportion of blue water footprint increased year by year with the passage of time, from 42.1% in 2000 to 47.5% in 2021, an increase of 12.6%. The increasing blue water footprint of wheat will inevitably put pressure on the sustainable use of regional water resources. The planting area of rice in Shanxi Province was small, and the blue water footprint and green water footprint decreased significantly during the study period (*p*<0.01), and the blue water footprint and green water footprint decreased by 59.9% and 64.4% respectively. The average blue water footprint of maize, wheat and rice from 2000 to 2021 were 61.1%, 38.7% and 0.2%, respectively. The proportion of blue water footprint of maize in the three grain crops increased significantly with time (*p*<0.01), and increased by 45.2% during the study period. The blue water footprint of wheat and rice decreased by 36.1% and 71.5%, respectively. On the basis that the availability of water resources in the region remains stable and the demand for water resources in the energy, livelihood and ecological sectors continues to increase (Liu et al., 2020). The continuous increase of blue water footprint of maize in Shanxi Province has put forward greater demand for irrigation water and brought new challenges to the balance of water supply and demand.

### Analysis of historical evolution characteristics of energy and water footprint

**Fig 5:**
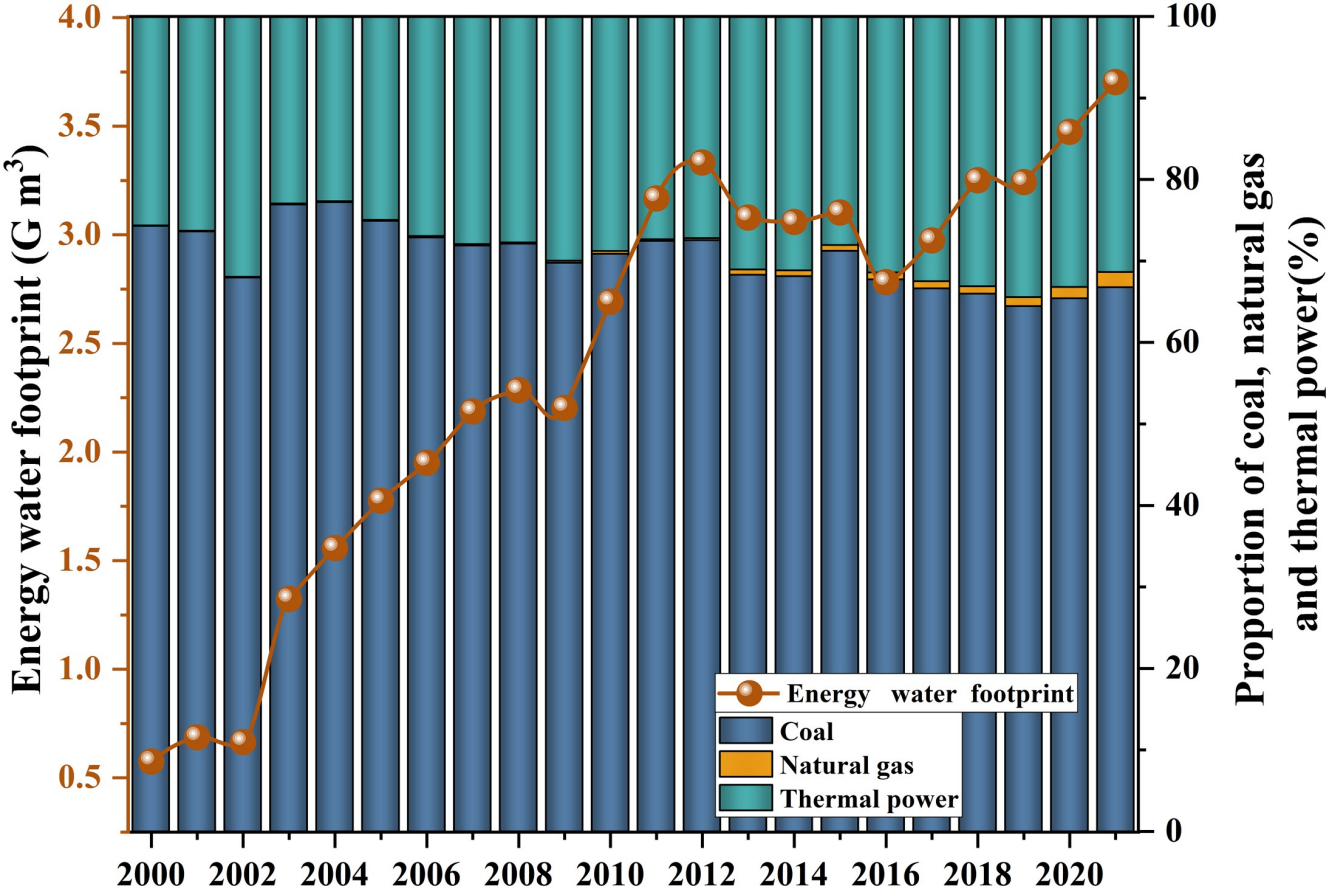
Trend of energy water footprint over time from 2000 to 2021.

The energy water footprint of Shanxi gradually increased over time. From 2000 to 2021, the energy water footprint showed a significant increase trend (*p*<0.01), with an average annual increase of 10.8% and an increase of 544.7% during the period (Fig 5). The water footprint of coal, natural gas and thermal power generation increased significantly over time (*p*<0.01). Compared with 2000, the water footprint of coal, natural gas and thermal power in 2021 increased by 2.7, 109.2 and 4.0 times, respectively. From the perspective of energy water footprint structure, the average proportion of coal, natural gas and thermal power generation water footprint were 70.6%, 0.5% and 28.9%, respectively. It can be seen that the three main energy industries in Shanxi were mainly coal and thermal power generation. Although the water footprint of coal, natural gas and thermal power generation has increased significantly over time, the energy structure has changed year by year, and the proportion of coal water footprint in energy water footprint was decreasing, while the proportion of natural gas and thermal power generation water footprint was increasing. The proportion of coal water footprint to energy water footprint decreased year by year with the passage of time, decreasing by 10.1% during the study period. The proportion of water footprint of natural gas and thermal power generation in energy water footprint increased year by year over time, increasing by 25.9 times and 22.3% respectively. The increasing of regional energy and water footprint will inevitably intensify the competition of various industries for water resources and bring greater pressure to regional water resources.

### Characterization of food-energy industry competition for water resources

**Fig 6:**
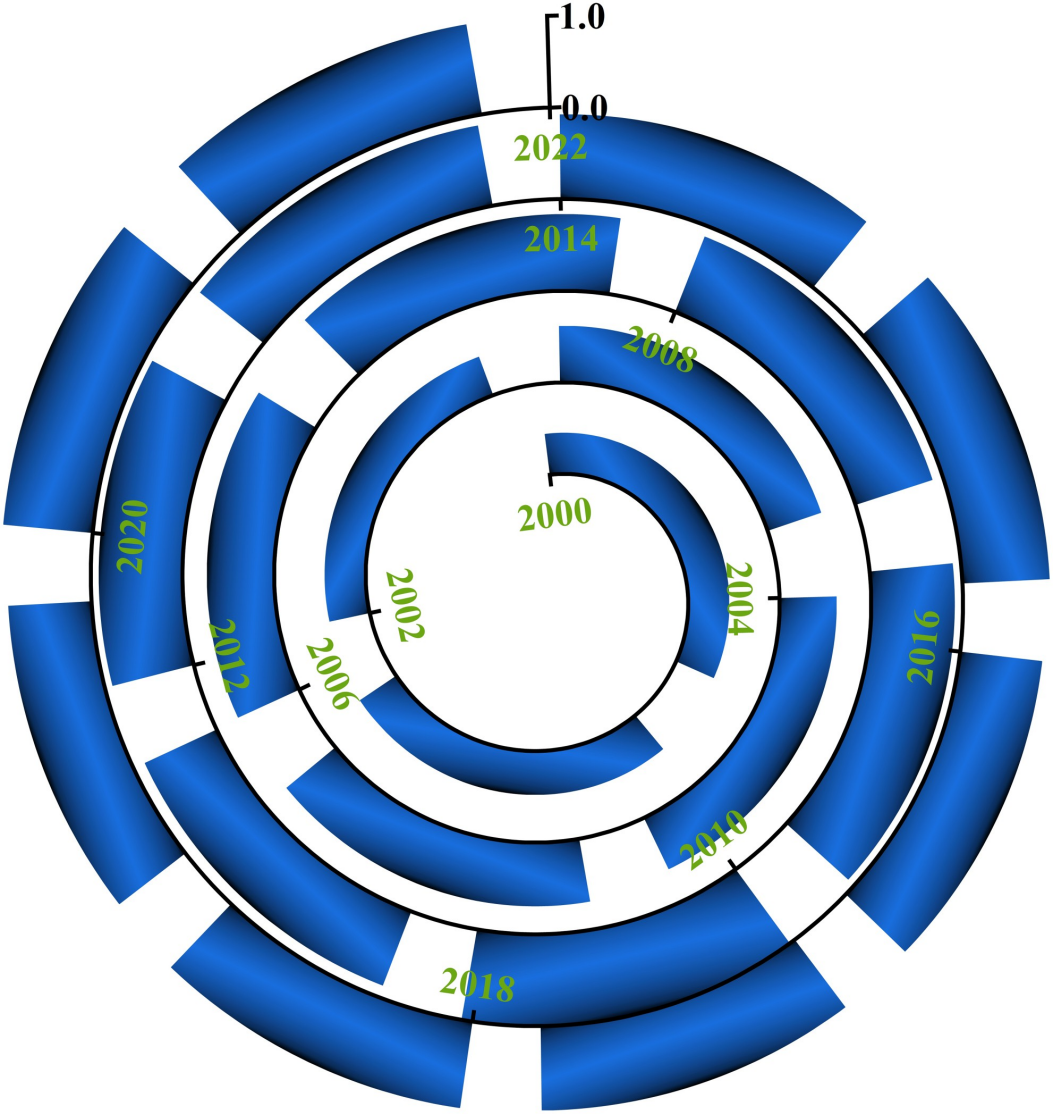
Competition index of food and energy industries for water resources.

The competition index of Shanxi grain and energy industry to water resources is increasing year by year. With the increase of regional grain water footprint and energy water footprint year by year, the competition index of food and energy industry for water resources has increased significantly. The average competition index of Shanxi’s food and energy industries for water resources from 2000 to 2021 was 0.77 (Fig 6). The competition index increased from 0.42 in 2000 to 0.94 in 2021, with an average annual growth rate of 4.3%. The increase in the competition index directly reflected the increasing competition for water resources in the development of regional food and energy industries. This will undoubtedly threaten the sustainable development of regional crops and energy industries, and bring greater challenges to ensuring regional food security and sustainable use of water resources.

## Discussion

With the continuous increase of China’s population and the acceleration of urbanization, the amount of food demanded by residents in the future will further increase, and the amount of water footprint required for food production will also increase significantly [32]. In addition, with the continuous expansion of China’s economic scale and the implementation of the western development strategy, the future demand for energy will also be further increased [42, 43]. As one of the regions with the most abundant coal resources in China, Shanxi plays an important role in ensuring China’s energy security. This is also destined to increase the amount of fossil energy exploitation in Shanxi in the future, resulting in further improvement of the energy and water footprint. Therefore, on the basis of the continuous increase of grain and energy water footprint, the regional food-energy industry will inevitably become more competitive for water resources in the future, which will threaten its healthy and sustainable development. Effective "throttling" measures must be taken in advance to alleviate regional water stress by reducing grain and energy water footprints.

### Effective measures to reduce grain water footprint

Crop production water footprint and grain yield are two direct factors affecting grain water footprint. Food production is closely related to the number of regional population and the demand for food. According to the existing research results, the population will further increase in the future, and the dietary structure will also change [32]. For the same amount of kcal provided, the production of animal products requires more raw grain and thus a larger water footprint [32]. Therefore, in order to ensure regional food security, it is not possible to reduce the grain water footprint and relieve the pressure of regional water resources by reducing regional grain production. The reduction of crop production water footprint helps to restrain the simultaneous increase of grain water footprint with the increase of grain yield. As shown in Fig 1, from 2000 to 2021, on the basis of an 81.1% increase in grain production, Shanxi Province’s grain water footprint only increased by 11.2% due to the reduction of crop production water footprint (38.5% decrease in 2021 compared with 2000). Similarly, for irrigation water, the blue water footprint of crop production decreased by 15.5%, which resulted in an increase of grain yield by 84.1% and an increase of grain blue water footprint by only 53.0%. Therefore, only by taking effective measures to improve the water use efficiency of crops and then achieve the purpose of reducing the water footprint of grain.

Recommended measures to reduce grain water footprint. (1) Optimizing the regional planting structure helps to reduce the water footprint of crop production. Due to the differences in regional natural conditions and agricultural production technology levels, the same crop has different production water footprints in different regions and has comparative advantages [31]. Similarly, in the same region, different crops have different production water footprints and have absolute comparative advantages [31]. Therefore, on the basis of not increasing agricultural production resources and technology input, by adjusting the regional planting structure, it is helpful to reduce the water footprint of regional grain crop production and reduce the amount of water resources needed in the process of grain production [31]. (2) By improving agricultural infrastructure and promoting water-saving irrigation techniques, it helps to reduce the water footprint of crop production. Accelerate the progress of farmland water conservancy infrastructure construction, increase effective irrigation area, improve channel anti-seepage technology, promote the transformation of channel water supply to pipeline water transmission, and then improve the utilization efficiency of crop water resources [44]. Vigorously promote the application of water-saving irrigation technology in farmers, and fundamentally change from traditional flood irrigation to precision irrigation [45], deficit irrigation [46, 47], drip irrigation [48], sprinkler irrigation [49], Bubbled-root irrigation [50], mulch irrigation [51] and other efficient water-saving or water-fertilizer integrated irrigation technology changes, greatly improving the water use efficiency of crops [52]. These measures can help to reduce the water footprint of crop production, which in turn reduces the food water footprint while maintaining food production and alleviating regional water stress. (3) Promote food virtual water trade within and outside the region, and indirectly achieve the purpose of reducing the amount of food water footprint demand within the region [53]. For example, Egypt saved 3.6 Gm^3^/year of national water resources by importing wheat [54]. Therefore, it is necessary to strengthen the grain trade in Shanxi Province and water-rich areas, and to alleviate the pressure of water resources in the region by importing water-intensive crop products. (4) Measures should be taken to improve the utilization efficiency of rainwater by crops, thereby indirectly reducing the demand for water resources for regional food production and reducing the blue water footprint [55]. For example, micro-rain planting technology (terrace + water cellar + drought-resistant crop combination planting technology, etc.), mechanized deep tillage and other technologies can be promoted in the field to improve the utilization efficiency of crops to precipitation [33, 56, 57]. The underground continuous seepage reservoir is established by making full use of the residual water and atmospheric precipitation in the flood season of rivers [58]. In addition, according to the characteristics of multi-year rainfall in the region, the use efficiency of green water by crops can be improved by adjusting the sowing date of crops [59]. (5) Establish a water-saving compensation mechanism to stimulate farmers’ water-saving driving force to a greater extent. Due to most area of China is still a traditional small-scale peasant production model, farmers need to invest more in water-saving irrigation facilities on the basis of water-saving irrigation, but farmers can’t obtain the social and environmental benefits generated by water-saving irrigation. This leads to the lack of enthusiasm of farmers to adopt water-saving irrigation technology for field management, which is also the resistance to the promotion of agricultural mechanization in China. Therefore, the government should formulate relevant policies in combination with factors such as water resources endowment, economic benefits, and the degree of competition of various industries for water resources in the region, and carry out a reasonable water-saving compensation mechanism for farmers. In addition, the government can also formulate high irrigation water prices, which is also an effective way to protect regional water resources [60]. Throughout the world, countries with higher agricultural water use efficiency have higher agricultural water prices, which is also a promotion measure to promote farmers’ agricultural reform [61]. The promotion of agricultural mechanization should also be accelerated, change the current planting mode of small-scale planting and fragmented farmland in China, and develop large-scale, mechanized and intelligent agriculture to solve the problem of low efficiency of agricultural water use [62].

### The severe challenges facing the future food-energy-water resources relationship

With a growing and increasingly affluent population, it is foreseeable that the region’s demand for food and energy will continue to increase in the future [32]. In order to meet the future residents’ demand for food and energy products, this often ignores the huge environmental costs they bring. For example, more water resources, land type changes and environmental pollution are needed. As the main energy industry in Shanxi, the corresponding water footprint of coal has an important impact on the size of the regional energy water footprint (Fig 5). The production process of coal not only leads to higher greenhouse gas emissions, but also a corresponding high water footprint [5]. According to the difference of coal mining mine (underground mine and open pit mine), lead to the difference of coal water footprint. Generally, due to the complexity of coal mining in underground mines, the water footprint is higher than that of open-pit mines. In all aspects of coal mining, coal washing is a process with large water consumption, which requires about 3.79–7.58 L/GJ [63]. Coal washing is to separate raw coal with different components and different proportions into different grades through the impact of water flow, remove dust and waste rock, and reduce ash and sulfur content. This process aims to improve combustion efficiency by reducing sulfur and particulate emissions during combustion to meet environmental standards [64]. Water can transport coal in the form of mud through pipelines [63]. In view of the large demand for water resources in the coal washing stage, the new coal washing technology can be popularized and applied. For example, dry coal gangue separation equipment, which can separate coal from coal gangue without water. The application of this technology can effectively reduce the competition of energy and food industry for water resources in water shortage areas, and effectively alleviate the pressure of regional water resources. Traditional fossil energy not only has a large demand for water resources, but also increases the concentration of carbon dioxide in the atmosphere. As a result, measures could be taken to increase renewable energy sources to replace fossil energy consumption, which would help to reduce the rate of fossil fuel depletion and carbon dioxide emissions in the region [5, 65, 66].

Under the condition of current production technology level, Shanxi’s grain production still has great development potential compared with China and even the global average [32]. Therefore, by increasing the input of crop production technology, technological innovation and agricultural ecologically efficient farming systems, the level of crop production can be effectively improved. In addition, some methods can alleviate the vicious competition of food and energy systems for water resources to a certain extent. The Chinese government should vigorously promote the energy industry and the consumption revolution [67], promote the high-quality development of the Yellow River Basin [68], and strengthen the inter-basin physical water transfer and food virtual water trade projects [69]. Measures should also be taken to strengthen agricultural production technology investment, improve crop production level and water resources utilization efficiency [70, 71]. In addition, the recovery, treatment and reuse of regional wastewater should also be strengthened [72–74]. In water-deficient areas, wastewater provides a potential alternative source of irrigation in some cases. This is of great significance for alleviating the competitive pressure of the future regional food and energy industry on water resources and better promoting the healthy and sustainable development of the region.

## Conclusions

Exploring the mechanisms of the regional food and energy industries compete for water resources, and then proposing effective countermeasures, will help to promote healthy and sustainable development in the region. This study focuses on the competitive mechanisms of food and energy industries for water resources in Shanxi Province, China, with a view to providing a reference for promoting the harmonious development of food-energy-water resources in other regions of China and the world. The main conclusions obtained in this paper are as follows:

As China’s demand for food security increases, grain production in China’s Shanxi Province is also increasing dramatically, exacerbating the amount of water demanded for regional grain production. The blue, green and gray water footprints of food are showing a significant increasing trend, which poses a challenge to sustainable regional food production. At the same time, as China’s demand for energy increases, the energy water footprint of Shanxi, a major coal province in China, has shown a significant increase. The water footprints of coal, natural gas and thermal power generation all show significant increasing trends, and the increase in energy water footprints exacerbates regional water resource pressures. As the food and energy industries demand increasing quantities of regional water resources, they will inevitably increase competition for the region’s limited water resources. The increase in the competition index is a direct reflection of the increasing competition for water resources in the development of the regional food and energy industries. This undoubtedly poses a threat to the sustainability of regional crop and energy industries. With the future strategic requirements of the government of China for food and energy security, the regional demand for food and energy security will be even more demanding, posing greater challenges to guaranteeing regional food security as well as the sustainable use of water resources. In order to better promote the healthy and sustainable development of the region, the mechanism of the impact of the food and energy industries on water resources is taken as an entry point, and suggested measures are proposed to reduce the number of regional crop and energy water footprints demanded, which will in turn better mitigate the intensity of competition for water resources by the regional food and energy industries and promote the benign development of the region. The relevant research results can provide theoretical references for other regions around the globe.

## Supporting information

S1 Table (CSV)

S2 Table (CSV)

S3 Table (CSV)

S4 Table (CSV)
